# miR-181b functions as an oncomiR in colorectal cancer by targeting PDCD4

**DOI:** 10.1007/s13238-016-0313-2

**Published:** 2016-09-19

**Authors:** Yanqing Liu, Uzair-ur-Rehman, Yu Guo, Hongwei Liang, Rongjie Cheng, Fei Yang, Yeting Hong, Chihao Zhao, Minghui Liu, Mengchao Yu, Xinyan Zhou, Kai Yin, Jiangning Chen, Junfeng Zhang, Chen-Yu Zhang, Feng Zhi, Xi Chen

**Affiliations:** 1 State Key Laboratory of Pharmaceutical Biotechnology, Collaborative Innovation Center of Chemistry for Life Sciences, Jiangsu Engineering Research Center for MicroRNA Biology and Biotechnology, NJU Advanced Institute for Life Sciences (NAILS), School of Life Sciences, Nanjing University, 163 Xianlin Road, Nanjing, 210046 China; 2 Modern Medical Research Center, Third Affiliated Hospital of Soochow University, 185 Juqian Road, Changzhou, 213003 China; 3 Department of Gastrointestinal Surgery, Nanjing Drum Tower Hospital Clinical College of Nanjing Medical University, 321 Zhongshan Road, Nanjing, 210008 China

**Keywords:** microRNA, colorectal cancer, miR-181b, PDCD4

## Abstract

Programmed cell death 4 (PDCD4) is a RNA-binding protein that acts as a tumor suppressor in many cancer types, including colorectal cancer (CRC). During CRC carcinogenesis, PDCD4 protein levels remarkably decrease, but the underlying molecular mechanism for decreased PDCD4 expression is not fully understood. In this study, we performed bioinformatics analysis to identify miRNAs that potentially target PDCD4. We demonstrated miR-181b as a direct regulator of PDCD4. We further showed that activation of IL6/STAT3 signaling pathway increased miR-181b expression and consequently resulted in downregulation of PDCD4 in CRC cells. In addition, we investigated the biological effects of PDCD4 inhibition by miR-181b both *in vitro* and *in vivo* and found that miR-181b could promote cell proliferation and migration and suppress apoptosis in CRC cells and accelerate tumor growth in xenograft mice, potentially through targeting PDCD4. Taken together, this study highlights an oncomiR role for miR-181b in regulating PDCD4 in CRC and suggests that miR-181b may be a novel molecular therapeutic target for CRC.

**Electronic supplementary material:**

The online version of this article (doi:10.1007/s13238-016-0313-2) contains supplementary material, which is available to authorized users.

## Introduction

Colorectal cancer (CRC) is a major worldwide health problem due to its high prevalence and mortality rate. In the USA, CRC is currently the third most common cancer type and the third leading cause of cancer-related death (Siegel et al., 2015). Although there has been a recent increase in the understanding, diagnosis and treatment of CRC, the exact mechanisms contributing to the origin and development of CRC remain complex and unclear.

In general, CRC results from an accumulation of genetic and epigenetic changes in colon epithelial cells. Among the myriad causes of CRC, oncogene activation (e.g., KRAS and IGF1R) and tumor suppressor gene silencing (e.g., APC and RET) play vital roles during tumorigenesis (Downward, 2003; Luo et al., 2013; Su et al., 2014; Dow et al., 2015). In recent years, programmed cell death 4 (PDCD4) has received considerable attention as a tumor suppressor protein (Biyanee et al., 2015). PDCD4 is a nuclear-cytoplasmic shuttling and RNA-binding protein involved in controlling the translation of specific mRNAs. Recent findings indicate that PDCD4 can function as a protein translation suppressor though both eIF4A-dependent or -independent pathways (Yang et al., 2003; Yang et al., 2004; Singh et al., 2011; Wedeken et al., 2011). PDCD4 is widely recognized as an important tumor suppressor, as its expression is dramatically downregulated in many cancer types, including CRC (Yang et al., 2006), lung cancer (Chen et al., 2003), breast cancer (Gonzalez-Villasana et al., 2012) and gastric cancer (Guo et al., 2013). Several studies have validated that loss of PDCD4 during tumorigenesis promotes cancer cell proliferation (Li et al., 2014), metastasis (Yang et al., 2006) and inhibits apoptosis (Wang et al., 2010b). Although our knowledge about PDCD4 has quickly increased in recent years, there is still much work to be done to elucidate how PDCD4 expression is regulated during carcinogenesis.

MicroRNAs (miRNAs) are small (19–23 nucleotides) non-coding RNA molecules (He and Hannon, 2004). In general, miRNAs act as endogenous suppressors of gene expression by binding to the 3′-untranslated region (3′-UTR) of target mRNAs to induce translational repression or mRNA cleavage (Bushati and Cohen, 2007). Since their discovery, miRNAs have gained attention as post-transcriptional regulators in many biological processes, including cancer (Esquela-Kerscher and Slack, 2006). Years of continued research on miRNAs have revealed that many miRNAs directly correlate with human cancers and that these miRNAs can function as tumor suppressor miRNAs or oncomiRs (Esquela-Kerscher and Slack, 2006). Abnormal miRNA expression also plays a pivotal role during CRC initiation, and some miRNAs directly regulate CRC cell proliferation, invasion and apoptosis. Among the miRNAs correlated with tumorigenesis, miR-181b is one of the most important. miR-181b was reported to be significantly overexpressed in CRC (Nakajima et al., 2006; Xi et al., 2006; Schetter et al., 2008; Degagne et al., 2014; Liu et al., 2014) and is associated with the poor prognosis of CRC patients (Schetter et al., 2008), indicating that miR-181b may be involved in the pathogenesis of CRC as an oncogene. Conversely, miR-181b has been found to be frequently downregulated in CRC samples due to higher hypermethylation and exert tumor-suppressive effects (Zhao et al., 2016). Thus, whether miR-181b functions as an oncogene or a tumor suppressor is dependent on the cell and tumor types, and miR-181b may exert different functions under different circumstances. Overall the findings of ours and others highlight distinguishing characteristics of miR-181b in both promoting and suppressing colorectal tumorigenesis. Thus, the molecular mechanism underlying the contribution of miR-181b to CRC development and progression needs to be fully elucidated.

STAT3, a transcription factor activated by IL-6, is closely associated with CRC (Morikawa et al., 2011; Pradhan et al., 2012; Waldner et al., 2012). Increased IL-6 expression has been correlated with advanced disease stage and decreased survival of CRC patients, and several therapeutics targeting the IL6/STAT3 signaling pathway have been developed as a promising strategy for treatment of CRC (Waldner et al., 2012). It is important to note that a large part of the functions of IL-6/STAT3 pathway resulted from their abilities to regulate downstream miRNAs (Iliopoulos et al., 2010; Degagne et al., 2014). One interesting example is that STAT3 directly activates transcription of miR-21 and miR-181b during the transformation process (Iliopoulos et al., 2010). Thus, investigating the functions of the downstream miRNAs of the IL6/STAT3 signaling pathway may contribute to our understanding of the molecular mechanisms underlying CRC development.

Although PDCD4, miR-181b and IL6/STAT3 signaling pathway are tightly associated with CRC carcinogenesis, it is necessary to explore their relationship and uncover the regulatory network constituted by them. In this study, we found that miR-181b, as an IL-6/STAT3-activated miRNA, directly targets PDCD4 to promote CRC cell proliferation and migration and to inhibit apoptosis *in vitro* and accelerate tumor growth *in vivo*.

## RESULTS

### CRC tissues exhibit downregulation of PDCD4 protein but not mRNA

**Figure 1:**
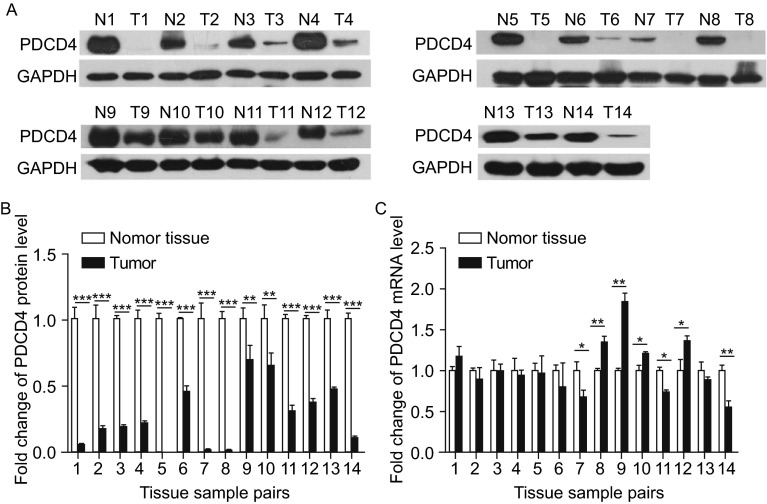
Downregulation of PDCD4 protein but not mRNA in CRC tissues. (A and B) Western blot analysis of PDCD4 protein levels in 14 paired CRC (T) and normal adjacent tissue (N) samples. A: representative images; B: quantitative analysis. (C) Quantitative RT-PCR analysis of PDCD4 mRNA levels in 14 paired CRC and normal adjacent tissue samples. *P < 0.05; **P < 0.01; ***P < 0.001

We first examined PDCD4 expression patterns in human CRC tissues. After measuring PDCD4 protein levels in 14 pairs of CRC tissues and normal adjacent tissues, we found that PDCD4 protein levels were dramatically decreased in CRC tissues compared to normal adjacent tissues (Fig. 1A and 1B). We then measured PDCD4 mRNA levels in the same CRC tissue pairs and detected irregular alterations in PDCD4 mRNA levels between the tumor and paired non-tumor tissues (Fig. 1C). The inconsistency between PDCD4 protein and mRNA expression in CRC tissues suggests that PDCD4 expression is regulated through a post-transcriptional mechanism.

### Identification of PDCD4 as a miR-181b target

**Figure 2:**
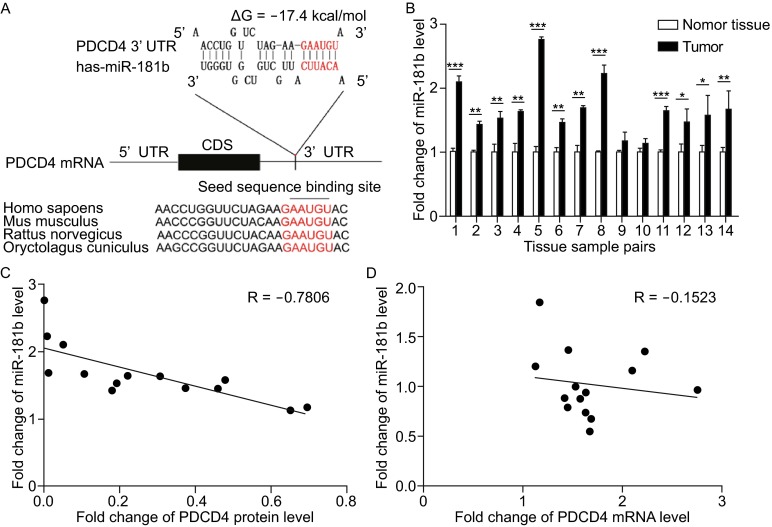
Identification of PDCD4 as a miR-181b target. (A) Schematic description of the hypothetical duplex formed by the interaction between the binding site in the PDCD4 3′-UTR (top) and miR-181b (bottom). The miR-181b seed region and the seed sequence of its binding site in the PDCD4 3′-UTR are indicated in red. All nucleotides of the seed sequence of the binding site are conserved in several species, including human, mouse and rat. The predicted free energy value of the hybrid is indicated. (B) Quantitative RT-PCR analysis of miR-181b expression levels in the same 14 pairs of CRC and normal samples. (C) Pearson’s correlation scatter plot of the fold change of miR-181b and PDCD4 protein in human CRC tissue pairs. (D) Pearson’s correlation scatter plot of the fold change of miR-181b and PDCD4 mRNA in human CRC tissue pairs. *P < 0.05; **P < 0.01; ***P < 0.001

Because miRNA is an important and widely existing way to post-transcriptionally regulate gene expression, we hypothesized that some miRNAs regulated PDCD4 protein level in CRC. We used a bioinformatics software Targetscan to predict potential miRNAs that target PDCD4. We identified miRNAs in the miR-181 family as the top candidates. There are four miR-181 family members (miR-181a/b/c/d) encoded by three independent transcripts on three separate chromosomes. Among them, human miR-181a/b have more gene copies (Ji et al., 2009) and higher expression levels (Landgraf et al., 2007) than miR-181c/d. In addition, miR-181a/b have been more closely implicated in cancer than miR-181c/d, and have thus received more attention in the miRNA field (Mansueto et al., 2010; Wang et al., 2010a; Liu et al., 2014; Parikh et al., 2014). For miR-181a/b, the minimum free energy value of the hybrid between miR-181b and the conserved binding site on the PDCD4 3′-UTR is -17.4 kcal/mol and is lower than that of miR-181a (−16.2 kcal/mol), suggesting that miR-181b may bind more tightly to PDCD4 3′-UTR than miR-181a. Thus, we used miR-181b for further experimentation. The predicted interaction between miR-181b and PDCD4 3′-UTR is illustrated in Fig. 2A. There was perfect base-pairing between the seed region (the core sequence that encompasses the first 2–8 bases of the mature miRNA) and the cognate target, and the miR-181b binding sequence in the PDCD4 3′-UTR was highly conserved across species (Fig. 2A).

We next examined whether miR-181b expression levels inversely correlated with PDCD4 expression in CRC tissues. We measured miR-181b levels in the same 14 pairs of CRC tissues and corresponding normal tissues and found that the miR-181b levels were consistently higher in CRC tissues (Fig. 2B), which was consistent with the idea that miRNA levels are inversely correlated to the levels of their targets. We further illustrated the inverse correlation between miR-181b and PDCD4 protein levels (Fig. 2C) and the disparity between the miR-181b and PDCD4 mRNA levels (Fig. 2D) using Pearson’s correlation scatter plots. Therefore, we considered PDCD4 to be a miR-181b target based on computational prediction and the inverse correlation between miR-181b and PDCD4 protein levels in human CRC tissues.

### miR-181b directly regulates PDCD4 expression at the post-transcriptional level

**Figure 3:**
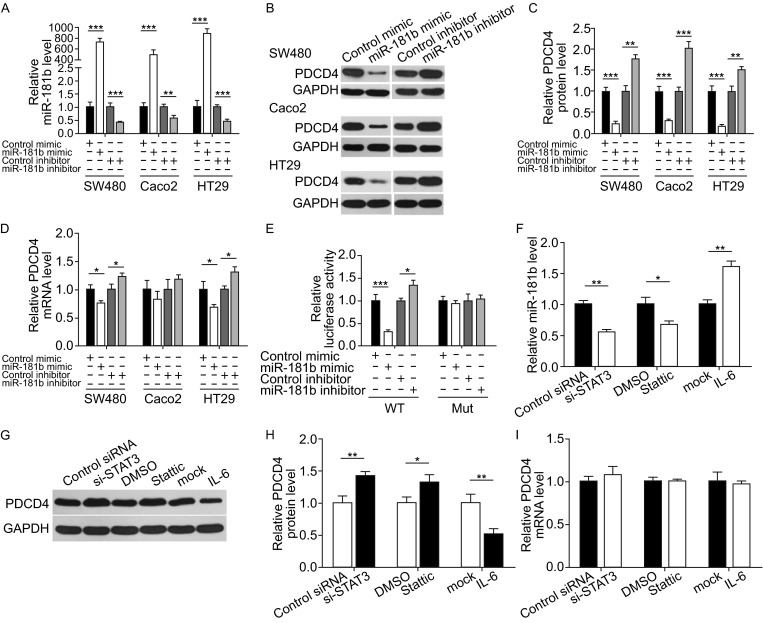
miR-181b directly regulates PDCD4 expression at the post-transcriptional level. (A) Quantitative RT-PCR analysis of miR-181b levels in SW480, Caco2 and HT-29 cells transfected with control mimic, miR-181b mimic, control inhibitor or miR-181b inhibitor. (B and C) Western blot analysis of PDCD4 protein levels in SW480, Caco2 and HT-29 cells transfected with control mimic, miR-181b mimic, control inhibitor or miR-181b inhibitor. B: representative images; C: quantitative analysis. (D) Quantitative RT-PCR analysis of PDCD4 mRNA levels in SW480, Caco2 and HT-29 cells transfected with control mimic, miR-181b mimic, control inhibitor or miR-181b inhibitor. (E) Direct binding of miR-181b to the PDCD4 3′-UTR. Firefly luciferase reporters containing the wild-type (WT) or mutant (MUT) form of human PDCD4 3′-UTR were cotransfected into SW480 cells along with control mimic, miR-181b mimic, control inhibitor or miR-181b inhibitor. At 24 h post-transfection, the cells were assayed using a luciferase assay kit. Firefly luciferase values were normalized to β-galactosidase activity and plotted as relative luciferase activity. For comparison, the luciferase activity in the control cells was set as 1. (F) Quantitative RT-PCR analysis of miR-181b levels in SW480 cells treated with control siRNA, STAT3 siRNA, DMSO, Stattic or IL-6. (G and H) Western blot analysis of PDCD4 protein levels in SW480 cells treated with control siRNA, STAT3 siRNA, DMSO, Stattic or IL-6. G: representative images; H: quantitative analysis. (I) Quantitative RT-PCR analysis of PDCD4 mRNA levels in SW480 cells treated with control siRNA, STAT3 siRNA, DMSO, Stattic or IL-6. *P < 0.05; **P < 0.01; ***P < 0.001

We further examined the inverse correlation between miR-181b and PDCD4 by evaluating PDCD4 expression levels in human CRC cells after miR-181b overexpression or knockdown. Fig. 3A demonstrates efficient miR-181b overexpression or knockdown in SW480 cells. As anticipated, PDCD4 protein expression significantly decreased upon miR-181b overexpression, whereas treatment with the miR-181b inhibitor increased PDCD4 protein levels in SW480 cells (Fig. 3B and 3C). We also examined PDCD4 mRNA expression after transfection. miR-181b overexpression or knockdown only slightly affected PDCD4 mRNA levels (Fig. 3D). To demonstrate the robustness of the test, we repeated the above experiments in two other CRC cell lines (Caco2 and HT29) and observed consistent results (Fig. 3A–D).

To determine whether miR-181b suppresses PDCD4 expression through direct interaction with the binding site in the PDCD4 3′-UTR, we fused the PDCD4 3′-UTR sequence containing the presumed miR-181b binding site into the downstream region of the firefly luciferase gene in a reporter plasmid. We transfected the resulting plasmid into SW480 cells along with the miR-181b mimic, miR-181b inhibitor or scrambled negative control RNAs. As expected, miR-181b overexpression resulted in an approximately 70% reduction in luciferase reporter activity compared to cells transfected with control mimic, whereas miR-181b inhibition resulted in a 30% increase in reporter activity compared to cells transfected with the control inhibitor (Fig. 3E). We next constructed a mutant plasmid by introducing point mutations into the miR-181b binding site in the PDCD4 3′-UTR to eliminate miR-181b binding ability. The mutated luciferase reporter was not affected by miR-181b overexpression or knockdown (Fig. 3E). These results suggest that the binding site strongly contributes to the miRNA-mRNA interaction.

It has been previously reported that the CRC-related transcription factor STAT3 (Morikawa et al., 2011; Pradhan et al., 2012) can activate miR-181b (Iliopoulos et al., 2010; Degagne et al., 2014). If PDCD4 is truly a miR-181b target, the alteration of STAT3 levels would simultaneously affect PDCD4 expression through miR-181b. To confirm this hypothesis, we used a STAT3 siRNA and a STAT3-specific inhibitor Stattic (Schust et al., 2006) to suppress STAT3 expression and activity, respectively, and used IL-6 to activate STAT3 activity (Iliopoulos et al., 2010). As anticipated, STAT3 siRNA and Stattic reduced miR-181b expression in CRC cells, while IL-6 treatment increased miR-181b expression (Fig. 3F). As a result, PDCD4 protein expression exhibited an inverse alteration to STAT3 (Fig. 3G and 3H). We also excluded the possibility that STAT3 is a direct regulator of PDCD4 by measuring the mRNA level of PDCD4 after the manipulation of the level or activity of STAT3. PDCD4 mRNA levels were not affected by STAT3 siRNA, Stattic (Schust et al., 2006) and IL-6 treatment (Fig. 3I), suggesting that STAT3 might not directly inhibit PDCD4 expression at the transcriptional level.

### miR-181b promotes CRC cell proliferation and migration and suppresses apoptosis by targeting PDCD4

**Figure 4:**
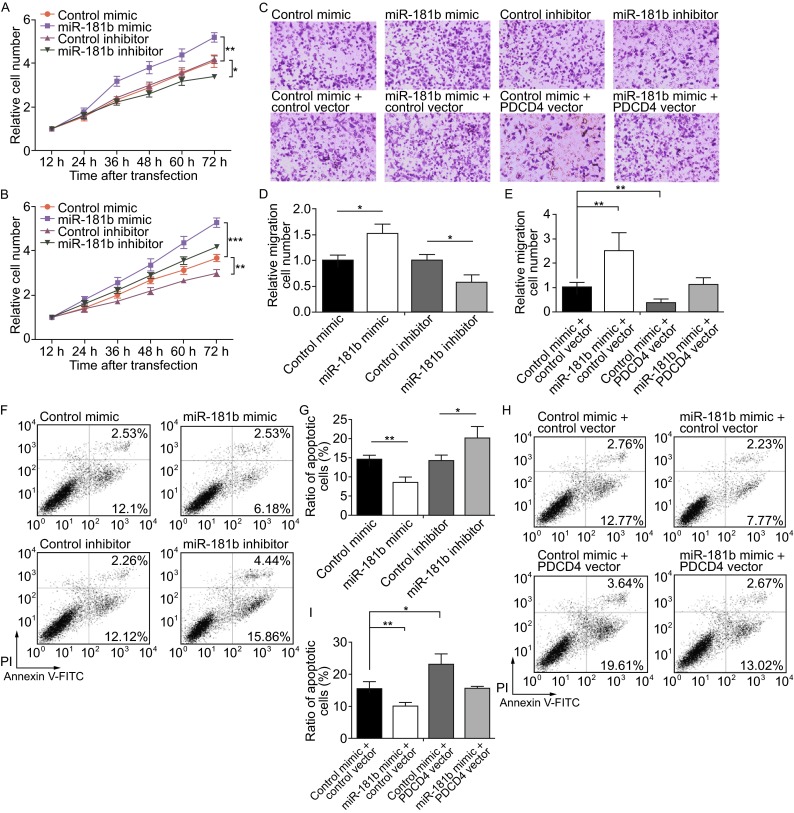
Effects of miR-181b and PDCD4 on CRC cell proliferation, migration and apoptosis. (A) Cell proliferation assays were performed 12, 24, 36, 48, 60 and 72 h after the transfection with equal dose of control mimic, miR-181b mimic, control inhibitor or miR-181b inhibitor. (B) Cell proliferation assays were performed 12, 24, 36 and 48, 60 and 72 h after transfection with equal dose of the control mimic plus control plasmid, miR-181b mimic plus control plasmid, control mimic plus PDCD4 overexpression plasmid, or miR-181b mimic plus PDCD4 overexpression plasmid. (C–E) Transwell analysis of SW480 cells that were transfected with equal dose of control mimic, miR-181b mimic, control inhibitor or miR-181b inhibitor (upper panel), or with equal dose of control mimic plus control plasmid, miR-181b mimic plus control plasmid, control mimic plus PDCD4 overexpression plasmid, or miR-181b mimic plus PDCD4 overexpression plasmid (lower panel). C: representative images; D and E: quantitative analysis of the number of migrated cells. (F and G) Apoptosis assays were performed 24 h after transfection of SW480 cells with equal dose of control mimic, miR-181b mimic, control inhibitor or miR-181b inhibitor. F: representative images; G: quantitative analysis of the apoptotic cell ratio. (H and I) Apoptosis assays were performed 24 h after transfection of SW480 cells with equal dose of the control mimic plus control plasmid, miR-181b mimic plus control plasmid, control mimic plus PDCD4 overexpression plasmid, or miR-181b mimic plus PDCD4 overexpression plasmid. H: representative images; I: quantitative analysis of the apoptotic cell ratio. *P < 0.05; **P < 0.01; ***P < 0.001

We hypothesized that miR-181b promotes the CRC oncogenic process by inhibiting PDCD4 expression. Thus, we investigated the effects of miR-181b-driven PDCD4 repression on CRC cell proliferation, migration and apoptosis. We first performed CCK-8 and transwell assays to analyze the effect of miR-181b on CRC cell proliferation and migration. SW480 cells transfected with miR-181b mimic exhibited increased proliferation and migration; in contrast, miR-181b inhibition had the opposite effect on cell proliferation and migration (Fig. 4A, 4C and 4D). We also investigated the effects of miR-181b on apoptosis by flow cytometric analysis. The percentage of apoptotic cells was significantly lower in SW480 cells transfected with miR-181b mimic and higher in cells transfected with miR-181b inhibitor compared to control cells (Fig. 4F and 4G).

However, it should be noted that transient transfection of miRNA mimics typically gives rise to supraphysiological miRNA levels in cells when the miRNA level is assessed by qRT-PCR (Thomson et al., 2013). The supraphysiological levels of transfected miRNA do not represent the functional levels, because the majority of transfected miRNA is not accessible for loading into Argonaute as functionally active miRNAs, but is actually in non-functional locations such as in lysosomes (Thomson et al., 2013). Thus, we constructed a lentivirus to produce functional intracellular miR-181b via the endogenous miRNA processing pathway. As shown in Supplemental Fig. 2A, the expression levels of mature miR-181b were found to be 3–4-fold higher than the basal levels when SW480 cells were infected with miR-181b overexpression lentivirus. Such fold change is biologically and physiological relevant, because the altered miR-181b also inhibited PDCD4 expression in SW480 cells (Fig. S2B and S2C). Furthermore, overexpression of miR-181b by lentivirus also promoted cell proliferation and migration and inhibited cell apoptosis in SW480 cells (Fig. S2D–H), to the same degree as those obtained by using miR-181b mimic. The results again verify that miR-181b regulates PDCD4 expression and consequently affects cell proliferation, migration and apoptosis.

Because a single miRNA can target hundreds of genes (Bartel, 2004), it is necessary to determine whether the effects of miR-181b on CRC cells are derived from miR-181b-mediated PDCD4 suppression. We next investigated the exact contribution of the miR-181b-PDCD4 axis on CRC cell proliferation, migration and apoptosis. We used an siRNA and an overexpression plasmid to respectively downregulate or upregulate PDCD4 protein levels. Efficient knockdown and overexpression of PDCD4 expression in SW480 cells is shown in Fig. S1A–C. As a result, PDCD4 downregulation activated proliferation and migration and suppressed apoptosis of SW480 cells, whereas PDCD4 overexpression had the opposite effect on cell proliferation, migration and apoptosis (Fig. S1D–I). Thus, miR-181b and PDCD4 have opposing effects on cell proliferation, migration and apoptosis in CRC. We subsequently investigated whether overexpression of miR-181b-resistant PDCD4 (PDCD4 ORF) was sufficient to rescue PDCD4 suppression by miR-181b and attenuated the pro-proliferation, pro-migration and anti-apoptotic effects of miR-181b on CRC cells. We transfected SW480 cells with miR-181b mimic or PDCD4 overexpression plasmid or co-transfected with miR-181b mimic and PDCD4 overexpression plasmid. As expected, cells co-transfected with the miR-181b mimic and PDCD4 overexpression plasmid showed significantly lower proliferation rates (Fig. 4B) and migration capabilities (Fig. 4C and 4E) and higher apoptosis rates (Fig. 4H and 4I) compared to cells transfected with miR-181b mimic alone. Thus, restoration of PDCD4 expression can reverse miR-181b-induced cell proliferation and migration and miR-181b-suppressed cell apoptosis, suggesting that targeting of PDCD4 is one mechanism by which miR-181b exerts its oncomiR function.

### miR-181b promotes CRC growth *in vivo* by targeting PDCD4

**Figure 5:**
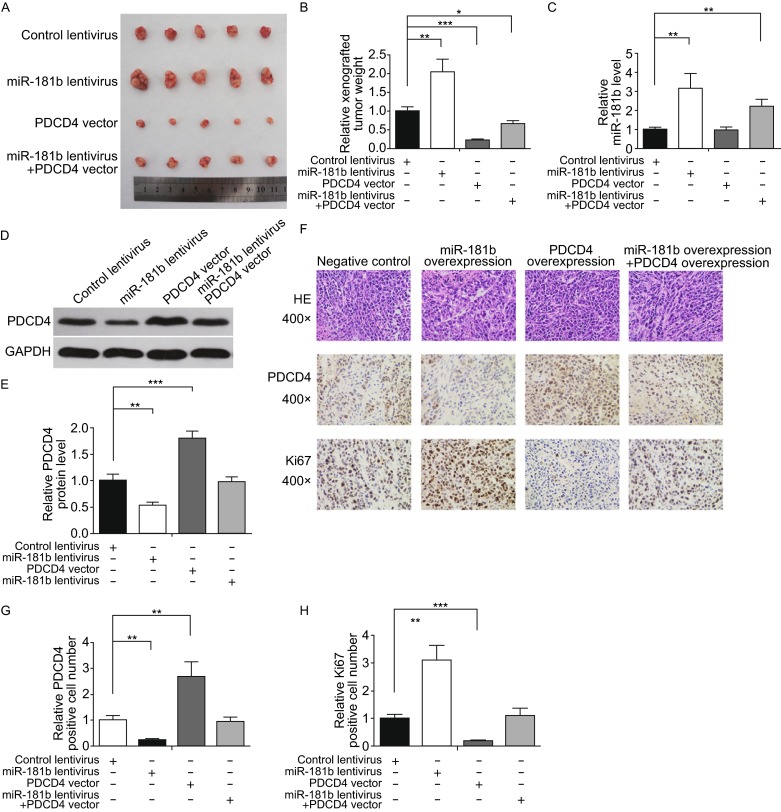
Effects of miR-181b and PDCD4 on the growth of CRC cell xenografted tumors in mice. SW480 cells were infected with a control lentivirus or a lentivirus to overexpress miR-181b, or transfected with a PDCD4 overexpression plasmid, or co-transfected with a miR-181b overexpression lentivirus and a PDCD4 overexpression plasmid. Differentially treated SW480 cells (3 × 10^6^ cells per mouse) were subcutaneously implanted into 4-week-old male SCID mice (5 mice per group), and tumor growth was evaluated 30 days after cell implantation. (A) Representative images of tumors from the implanted mice. (B) Quantitative analysis of xenografted tumor weights. (C) Quantitative RT-PCR analysis of miR-181b levels in tumors from implanted mice. (D and E) Western blotting analysis of PDCD4 protein levels in tumors from implanted mice. D: representative images; E: quantitative analysis. (F–H) H&E-stained sections and immunohistochemical staining for PDCD4 and Ki-67 in tumors from implanted mice. F: representative images; G and H: quantitative analysis. *P < 0.05; **P < 0.01; ***P < 0.001

Finally, we investigated the effects of miR-181b and PDCD4 on the growth of CRC xenografts in mice. We infected SW480 cells with a control lentivirus or a miR-181b overexpression lentivirus, transfected cells with a PDCD4 overexpression plasmid, or co-transfected with a miR-181b overexpression lentivirus and a PDCD4 overexpression plasmid. The effects of lentiviral infection and plasmid transfection are shown in Fig. S2A–C. Subsequently, we subcutaneously implanted the infected or transfected SW480 cells into 4-week-old nude mice. We evaluated tumor growth 30 days after cell implantation. Xenograft tumors from miR-181b-overexpressing group exhibited a significant increase in size and weight compared to the control group, whereas the sizes and weight of tumors in the group implanted with PDCD4-overexpressing cells dramatically decreased (Fig. 5A and 5B). Additionally, PDCD4 overexpression attenuated the growth-promoting effects of miR-181b (Fig. 5A and 5B), suggesting that miR-181b promotes tumor growth by silencing PDCD4. We next isolated and analyzed total RNA and protein from the tumors. Tumors from the miR-181b-overexpressing group showed a significant increase in mature miR-181b expression compared to tumors from the control group (Fig. 5C). Likewise, tumors from the miR-181b-overexpressing group expressed decreased PDCD4 protein levels compared to tumors from the control group, whereas tumors from the PDCD4-overexpressing group showed elevated PDCD4 protein levels (Fig. 5D and 5E). Moreover, tumors with both miR-181b and PDCD4 overexpression exhibited significantly higher PDCD4 levels compared to tumors overexpressing miR-181b alone (Fig. 5D and 5E), suggesting that PDCD4 overexpression rescued miR-181b-mediated PDCD4 suppression. We embed xenografted tumors in paraffin and then performed H&E staining or examined using immunohistochemical assays. H&E staining of xenograft tissues showed increased cell mitosis in the miR-181b lentivirus group and decreased mitosis in the PDCD4 plasmid group, whereas xenografts with both miR-181b and PDCD4 overexpression exhibited less cell mitosis compared to xenografts with miR-181b overexpression (Fig. 5F). Immunohistochemical staining also revealed lower PDCD4 levels in tumors from mice implanted with miR-181b-overexpressing cells, whereas tumors from the PDCD4-overexpressing mice showed increased PDCD4 protein levels (Fig. 5F and 5G). Finally, we assessed the proliferative activity of tumor cells via Ki-67 immunohistochemical staining. The percentage of Ki-67-positive tumor cells was increased in the group implanted with miR-181b lentivirus and decreased in the group implanted with PDCD4 plasmid (Fig. 5F and 5H). Likewise, PDCD4 overexpression attenuated the pro-proliferative effects of miR-181b overexpression (Fig. 5F and 5H). These results are consistent with the *in vitro* findings, which firmly validated the oncomiR role of miR-181b in CRC tumorigenesis through targeting of PDCD4.

## Discussion

Colorectal cancer is the third most common cancer worldwide. At the molecular level, colorectal cancer arises from a series of genetic and epigenetic alterations that inactivate tumor suppressor genes and activate oncogenes. The list of classical tumor suppressors and oncogenes has been expanded to include miRNAs. Many studies have reported the extensive alteration of miRNA profiles in the initiation and developmental stages of CRC (Brunet Vega et al., 2013; Xu et al., 2016). More importantly, new studies have established the potential usefulness of miRNAs as therapeutic molecules against cancer (Kota et al., 2009; Ma et al., 2010). For example, aberrantly activated miRNAs can be silenced using antagomirs (Krutzfeldt et al., 2005), and re-expression of miRNAs that are lost in cancers can be achieved by the induction of miRNA mimics (Kota et al., 2009). In this study, we showed that miR-181b was upregulated in CRC tissues and promoted cell proliferation and migration and suppressed cell apoptosis *in vitro* and accelerated tumor growth *in vivo*. Consistent with these results, miR-181 has also been reported to be upregulated in various human cancer types, including CRC (Nakajima et al., 2006; Xi et al., 2006; Schetter et al., 2008; Degagne et al., 2014; Liu et al., 2014), hepatocellular cancer (Wang et al., 2010a), breast cancer (Mansueto et al., 2010) and ovarian cancer (Parikh et al., 2014). During the CRC oncogenic process, miR-181 can promote tumor growth and metastasis (Ji et al., 2014) and is tightly associated with a poor prognosis (Nishimura et al., 2012) and the chemoresponse of CRC patients (Nakajima et al., 2006). Thus, it is possible that targeting of miR-181b could control CRC development and ameliorate the symptoms, as shown by other groups (Krutzfeldt et al., 2005; Kota et al., 2009; Ma et al., 2010). More research emphasis is required to characterize the feasibility of targeting miR-181b in CRC therapy and to develop simplified and cost-effective manipulation methods.

PDCD4 is an important tumor suppressor in many cancers. In this study, we demonstrated that PDCD4 was dramatically downregulated in CRC tissues and could inhibit proliferation and migration and promote apoptosis in CRC cells and attenuate tumor growth in xenografted mice. However, despite these advances in our understanding of the essential role of PDCD4 in cancer progression, the precise molecular mechanism through which PDCD4 is downregulated during tumorigenesis remains largely unknown. We searched for miRNAs that could target PDCD4 and experimentally validated miR-181b as a direct regulator of PDCD4. However, because a single miRNA can target multiple genes and multiple miRNAs can target a single gene, miR-181b may have multiple different targets in addition to PDCD4, and PDCD4 may be regulated by different miRNAs in addition to miR-181b. For example, PDCD4 is regulated by miR-21 (Li et al., 2014) and miR-183 (Yang et al., 2014), and miR-181b can simultaneously target WIF-1 (Ji et al., 2014) and CYLD (Iliopoulos et al., 2010). Therefore, it is important to investigate how critical this new pathway will be in the network of colorectal tumorigenesis pathways. Because restoration of PDCD4 expression attenuated the growth-promoting effects of miR-181b, targeting of PDCD4 may be a mechanism by which miR-181b exerts its oncomiR function. Therefore, modulation of PDCD4 by miR-181b may explain why miR-181b is aberrantly overexpressed and PDCD4 is weakly expressed in CRC tissues and why miR-181b upregulation can promote cell growth and CRC formation.

It is necessary to find out the upstream regulator of miR-181b in CRC. In this study, we showed that STAT3 suppressed PDCD4 through miR-181b activation. These findings add new clues to understanding the role of STAT3 in CRC: activation of IL-6/STAT3 suppressed PDCD4 by upregulating miR-181b, and therefore promoting the development of CRC. Consistent with these results, a recent study demonstrated that PDCD4 deficiency promoted the IL-6/STAT3 pathway in mice (Wang et al., 2016). Thus, this study delineates a novel regulatory network employing IL-6/STAT3, miR-181b and PDCD4 as an integrated feedback loop to fine-tune cell function in colorectal cells. This signaling pathway is crucial for normal colorectal cell function, and any disruption in this pathway may result in misbehavior of colorectal cells and CRC formation.

In summary, this study identified for the first time that miR-181b can target PDCD4 to promote CRC tumorigenesis. Future research on miR-181b and PDCD4 will provide us more knowledge about CRC and pave new approaches for molecular therapeutics for this disease.

## Materials and Methods

### Tissue samples

CRC tissue and paired normal adjacent tissue samples were acquired from patients undergoing a surgical procedure at the Affiliated Drum Tower Hospital of Nanjing University Medical School (Nanjing, China). Both the tumor and non-tumor tissues were sent for histological analysis and diagnostic confirmation. Written consent was obtained from all patients, and all protocols concerning the use of patient samples in this study were approved by the Ethics Committee of Nanjing University. Tissue samples were immediately frozen in liquid nitrogen at the time of surgery and stored at -80°C. All experiments were performed in accordance with The Code of Ethics of the World Medical Association (Declaration of Helsinki) and approved guidelines of the Nanjing University. The clinical features of the patients are listed in Supplemental Table 1.

### Cell culture

The SW480, Caco2 and HT29 human CRC cell lines were purchased from the Shanghai Institute of Biochemistry and Cell Biology, Chinese Academy of Sciences (Shanghai, China). SW480 and HT29 cells were cultured in RPMI-1640 (Gibco, Carlsbad, CA, USA) supplemented with 10% fetal bovine serum (FBS, Gibco) in a humidified incubator at 37°C with 5% CO_2_. Caco2 cells were cultured in DMEM (Gibco) supplemented with 10% FBS (Gibco) in a humidified incubator at 37°C with 5% CO_2_.

### RNA isolation and quantitative RT-PCR

Total RNA was extracted from the cultured cells and human tissues using Trizol Reagent (Invitrogen, CA, USA) according to the manufacturer’s instructions. TaqMan miRNA Assay Probes (Applied Biosystems, Foster City, CA) were used to quantify mature miR-181b according to the manufacturer’s instructions. Briefly, 1 μg of total RNA was reverse-transcribed to cDNA using a stem-loop RT primer (Applied Biosystems) and AMV reverse transcriptase (TaKaRa, Dalian, China). The reaction conditions were as follows: 16°C for 30 min, 42°C for 30 min and 85°C for 5 min. Quantitative real-time PCR was performed using a TaqMan PCR kit on an Applied Biosystems 7500 Sequence Detection System (Applied Biosystems). The reactions were incubated in a 96-well optical plate at 95°C for 5 min, followed by 40 cycles of 95°C for 15 s and 60°C for 1 min. All of the reactions were run in triplicate. After the reactions were complete, the cycle threshold (C_T_) data were determined using fixed threshold settings, and the mean C_T_ was determined from triplicate PCRs. A comparative C_T_ method was used to compare each condition to the control reactions. U6 snRNA was used as an internal control, and the relative amount of miRNA normalized to U6 was calculated with the equation 2^-ΔΔCT^, in which ΔΔC_T_ = (C_T__miR-181b_ − C_T__U6_)_tumor_ − (C_T__miR-181b_ − C_T__U6_)_control_.

To quantify PDCD4 and GAPDH mRNA, 1 μg of total RNA was reverse transcribed to cDNA using Oligo d(T)18 primers (TaKaRa) and AMV reverse transcriptase (TaKaRa). The reaction conditions were as follows: 42°C for 60 min and 85°C for 5 min. Real-time PCR was performed using the RT product, SYBR Green dye (Invitrogen) and specific primers for PDCD4 and GAPDH. The primer sequences were as follows: PDCD4 (sense): GTTGGCAGTATCCTTAGCATTGG; PDCD4 (antisense): TCCACATCAGTTGTGCTCATTAC; GAPDH (sense): CGAGCCACATCGCTCAGACA; and GAPDH (antisense): GTGGTGAAGACGCCAGTGGA. The reactions were incubated at 95°C for 5 min, followed by 40 cycles of 95°C for 30 s, 60°C for 30 s and 72°C for 30 s. After the reactions were complete, the C_T_ values were determined by setting a fixed threshold. The relative amounts of PDCD4 mRNAs were normalized to GAPDH using a similar method as described above.

### Protein isolation and western blot

Cells or tissues were lysed in RIPA lysis buffer (Beyotime, Shanghai, China) with freshly added PMSF (Beyotime, Shanghai, China) for 30 min on ice and were centrifuged at 12,000 × g at 4°C for 10 min. The supernatant was collected, and the protein concentration was calculated using a BCA protein assay kit (Thermo Scientific, Rockford, IL, USA). Proteins were separated by SDS-PAGE (Bio-Rad). After electrophoresis, the proteins were electrotransferred to PVDF membranes (Roche) and then blocked with 5% skim milk for 1 h. The membranes were then incubated with primary antibodies at 4°C overnight. After 4 washes (10 min each) in TBST, the membranes were incubated with horseradish peroxidase-conjugated secondary antibody for 1 h at room temperature. After 4 washes (10 min each), the membranes were incubated with Pierce SuperSignal West Pico chemiluminescence substrate (Thermo). The same membrane was also probed with a GAPDH antibody as a control. Antibodies against PDCD4 and GAPDH were purchased from Santa Cruz Biotechnology (sc-130545 and sc-25778, Santa Cruz, CA, USA).

### miRNA overexpression and knockdown

miR-181b overexpression was achieved by transfecting CRC cells with a miR-181b mimic, a synthetic double-stranded RNA oligonucleotide mimicking the miR-181b precursor. miR-181b knockdown was achieved by transfecting CRC cells with miR-181b inhibitor, a chemically modified antisense oligonucleotide designed to target mature miR-181b. Synthetic miR-181b mimic and inhibitor and scrambled negative control RNAs (control mimic and inhibitor) were purchased from GenePharma (Shanghai, China). SW480, Caco2 and HT29 cells were seeded in 6-well plates and transfected using Lipofectamine 2000 (Invitrogen) on the following day when the cells were approximately 60%–80% confluent. For miR-181b overexpression experiments, 200 pmol of miR-181b mimic or control mimic were added to each well. For miR-181b knockdown experiments, 200 pmol of miR-181b inhibitor or control inhibitor were added to each well. At 6 h after transfection, the SW480 and HT29 cell medium was changed to RPMI-1640 supplemented with 2% FBS and the Caco2 cell medium was changed to DMEM supplemented with 2% FBS. The cells were harvested 48 h after transfection for total RNA or protein isolation.

### Plasmid construction and siRNA interference assay

Mammalian expression plasmids designed to specifically express the full-length open reading frame (ORF) of the human PDCD4 gene were purchased from Genescript (Nanjing, China). An empty plasmid served as a negative control (control plasmid). siRNAs designed to specifically silence PDCD4 or STAT3 were purchased from GenePharma (Shanghai, China). A scrambled siRNA served as a control. The siRNA sequences were as follows: si-PDCD4: GCGGAAAUGUUAAGAGAUU; si-STAT3: CCACUUUGGUGUUUCAUAA. The overexpression plasmids and siRNAs were transfected into CRC cells using Lipofectamine 2000 (Invitrogen) according to the manufacturer’s instructions. Total RNA and protein were isolated 48 h after transfection and were assessed by quantitative RT-PCR and western blot, respectively.

### STAT3 activation and inhibition

To activate STAT3, recombinant human IL-6 (200-06, PeproTech) was added to CRC cells at a final concentration of 80 ng/mL. To suppress STAT3 activity or expression, a STAT3-specific inhibitor Stattic (S7947, Sigma) or an siRNA against STAT3 (sequence described above) was added to CRC cells.

### Luciferase reporter assay

A 378-bp fragment of the PDCD4 3′-UTR containing the presumed miR-181b binding site was amplified by PCR with human genomic DNA as a template. The following set of primers (with restriction enzyme cut site and protection bases in the 5′ end) was used: *GGACTAGT*GCTGCTGCTGTTGAGATA (forward primer) and *CCCAAGCTT*CAAGGGAACACTAAGATTAA (reverse primer). The 378-bp fragment is located in the last exon of PDCD4. The two additional sequences *GGACTAGT* and *CCCAAGCTT* in each primer were specially added for restriction enzyme Spe I and Hind III, respectively. The PCR fragment was digested with Spe I and Hind III restriction enzyme and then inserted into the Spe I-Hind III sites of the pMIR-REPORT plasmid (Ambion, USA). Successful insertion was confirmed by DNA sequencing. To test binding specificity, sequences that interacted with the miR-181b seed sequence were mutated from GAATGT to CTTACA, and the synthetic PDCD4 3′-UTR mutant fragment was inserted into an equivalent reporter plasmid. For the luciferase reporter assays, SW480 cells were cultured in 24-well plates, and each well was co-transfected with 0.2 µg of firefly luciferase reporter plasmid, 0.2 µg of β-galactosidase (β-gal) expression plasmid (Ambion), and equal amounts (50 pmol) of miR-181b mimic, miR-181b inhibitor or the scrambled negative control RNAs using Lipofectamine 2000 (Invitrogen). The β-gal plasmid was used as a transfection efficiency control. The cells were assayed using a luciferase assay kit 24 h post-transfection (Promega, Madison, WI, USA).

### Cell proliferation assay

SW480 cells were plated at 2 × 10^4^ cells per well in 96-well plates and incubated overnight in RPMI-1640 supplemented with 10% FBS. The cell proliferation index was measured using the Cell Counting Kit-8 (CK04-500, Dojindo, Japan) at 12, 24, 36, 48, 60 and 72 h post-transfection according to the manufacturer’s instruction. Absorbance was measured at a wavelength of 450 nm. All experiments were performed in triplicate.

### Cell migration assay

Cell migration assays were performed using Millipore 24-Well Millicell (Millipore) plates containing an 8-μm pore membrane. The bottom face of the membrane was coated with 1% gelatin. Cells were harvested 24 h after transfection and suspended in FBS-free RPMI-1640 culture medium. The cells were then added to the upper chamber (2 ×10^4^ cells/well) and 0.5 mL RPMI-1640 plus 20% FBS was added to the lower compartment. The Transwell-containing plates were incubated for 24 h in the incubator. After incubation, cells that had entered the lower surface of the filter membrane were fixed with 4% paraformaldehyde for 25 min at room temperature. The membrane was washed 3 times with distilled water and stained with 0.1% crystal violet in methanol for 15 min at room temperature. Cells remaining on the upper surface of the filter membrane (non-migrant) were gently scraped off with a cotton swab. The lower surfaces (with migrant cells) were captured by photomicroscopy (BX51 Olympus, Japan), and the cells were blindly counted (five fields per chamber).

### Cell apoptosis assay

SW480 cell apoptosis was tested using an Annexin V-FITC/propidium iodide (PI) staining assay. SW480 cells were cultured in 6-well plates and transfected with the above-mentioned RNAs or plasmids. To induce apoptosis, LPS (Sigma) was added to a final concentration of 400 ng/mL. The cells were harvested after culturing for 24 h in FBS-depleted medium. Flow cytometric analysis of apoptotic cells was performed using an Annexin V-FITC/PI staining kit (BD Biosciences, CA, USA). After washing with cold PBS, the cells were resuspended in binding buffer (100 mmol/L HEPES, pH 7.4; 100 mmol/L NaCl; and 25 mmol/L CaCl_2_), followed by staining with Annexin V-FITC/PI at room temperature in the dark for 15 min. Apoptotic cells were then evaluated by gating the PI- and Annexin V-positive cells using a fluorescence-activated cell-sorting (FACS) flow cytometer (BD Biosciences, San Jose, CA, USA). All experiments were performed in triplicate.

### Establishment of tumor xenografts in mice

4-week-old male SCID (severe combined immune deficiency) mice (*nu*/*nu*) were purchased from the Model Animal Research Center of Nanjing University (Nanjing, China) and maintained under specific pathogen-free conditions at Nanjing University. A 300-bp fragment containing the miR-181b genomic sequence was obtained by PCR amplification of human DNA and cloned into a lentiviral expression vector. SW480 cells were infected with a control lentivirus or a miR-181b overexpression lentivirus, transfected with a PDCD4 overexpression plasmid, or co-transfected with a miR-181b overexpression lentivirus and a PDCD4 overexpression plasmid. After infection and/or transfection, SW480 cells were subcutaneously injected into SCID mice (3 × 10^4^ cells in 0.2 mL PBS per mouse, 5 mice per group). The needle was inserted into the left side of the armpit, midway down, 5 mm deep at a 45° angle. Mice were sacrificed 30 days after injection to remove the xenografted tumors, and the weights of mice and tumors were measured. A portion of the tissues was used for protein and total RNA extraction, and the remaining tissue was fixed in 4% paraformaldehyde for 24 h. The tissue was processed for Hematoxylin and Eosin (H&E) staining or immunohistochemical staining for PDCD4 and Ki-67. All experiments were approved by the Institutional Review Board of Nanjing University (Nanjing, China) and performed in accordance with the U.K. Animals (Scientific Procedures) Act (1986) and the guidelines of the National Institutes.

### Statistical analysis

All of the images of western blot assay and migration assay were representative of at least three independent experiments. Quantitative RT-PCR assay, luciferase reporter assay, proliferation assay and apoptosis assay were performed in triplicate, and each individual experiment was repeated several times. The results are presented as the means ±SE of at least three independent experiments. Observed differences were considered statistically significant at *P* < 0.05 using Student’s *t*-test.

## Electronic supplementary material

Below is the link to the electronic supplementary material.

Supplementary material 1 (PDF 724 kb)

## Abbreviations

CRC: Colorectal cancer
miRNA: microRNA
PDCD4: Programmed cell death 4
