# Supplementary material for: miR-181b functions as an oncomiR in colorectal cancer by targeting PDCD4
Source: Protein Cell. 2016 Sep 19;7(10):722–34. doi: 10.1007/s13238-016-0313-2 (PMC5055492; doi:10.1007/s13238-016-0313-2)
Supplement: Supplementary file 1 — Supplementary material 1 (PDF 724 kb) [file 13238_2016_313_MOESM1_ESM.pdf]

miR-181b functions as an oncomiR in colorectal cancer by targeting PDCD4

Supplemental Table 1. Clinical features of colorectal cancer patients.

Case number	Age	Gender	Pathological Stage	Tumor Subtype
1	58	M	II(T3,N0,M0)	Adenocarcinoma
2	73	M	II(T3,N1,M0)	Adenocarcinoma
3	65	F	III(T3,N2,M0)	Adenocarcinoma
4	63	M	III(T3,N1,M0)	Adenocarcinoma
5	67	F	II(T3,N0,M0)	Adenocarcinoma
6	59	F	III(T3,N1,M0)	Adenocarcinoma
7	71	F	III(T3,N1,M0)	Adenocarcinoma
8	46	M	III(T3,N0,M1)	Adenocarcinoma
9	56	M	II(T3,N0,M0)	Adenocarcinoma
10	58	F	II(T3,N0,M0)	Adenocarcinoma
11	43	M	III(T3,N1,M0)	Adenocarcinoma
12	60	M	III(T3,N1,M0)	Adenocarcinoma
13	55	F	II(T3,N0,M0)	Adenocarcinoma
14	43	F	III(T3,N1,M0)	Adenocarcinoma

Supplemental figure 1.

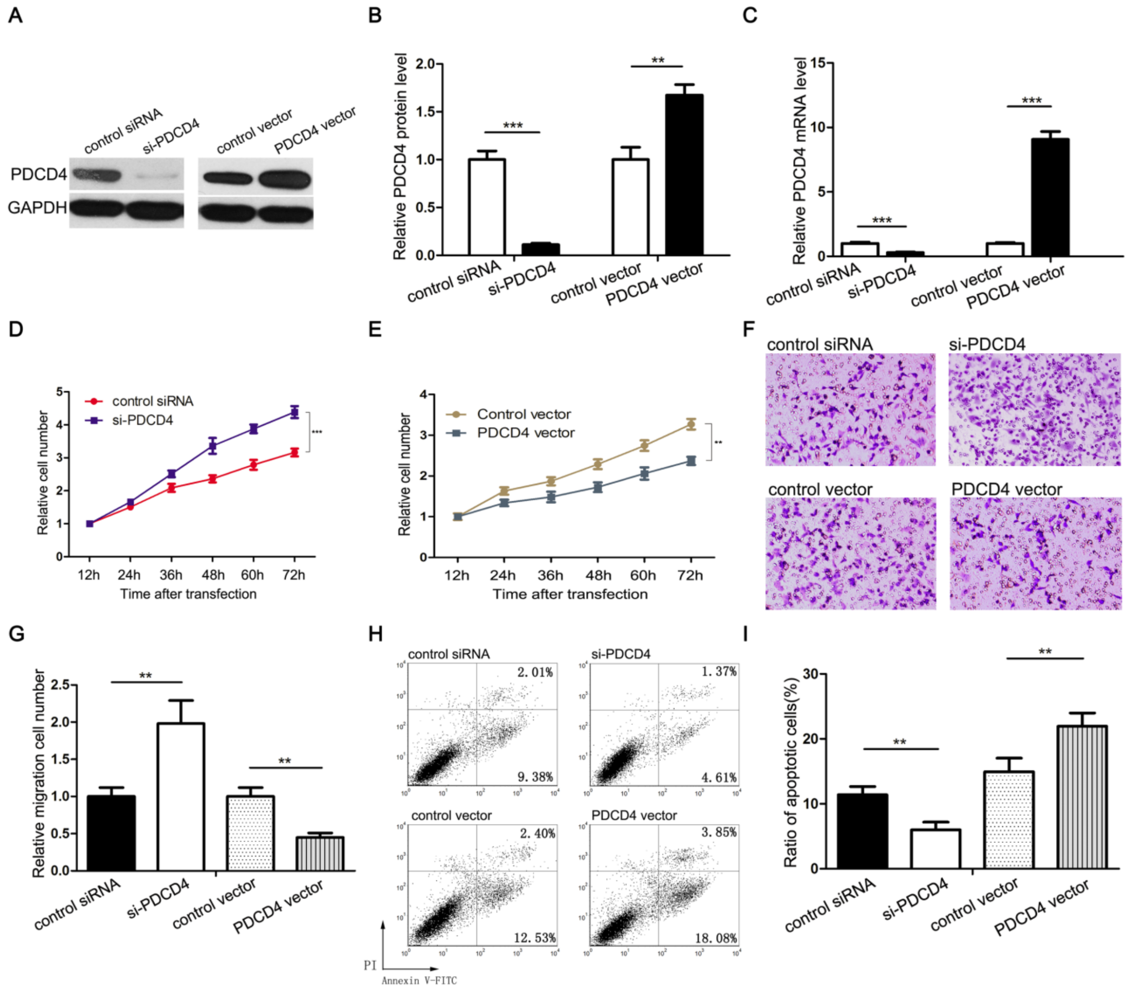

Supplemental Figure 1. Effect of PDCD4 on CRC cell proliferation, migration and apoptosis. **(A and B)** Western blotting analysis of PDCD4 protein levels in SW480 cells transfected with control siRNA, PDCD4 siRNA, control plasmid or PDCD4 plasmid. A: representative images; B: quantitative analysis. **(C)** Quantitative RT-PCR analysis of PDCD4 mRNA levels in SW480 cells transfected with control siRNA, PDCD4 siRNA, control plasmid or PDCD4 plasmid. **(D)** Cell proliferation assays were performed 12, 24, 36, 48, 60 and 72 h after the transfection of SW480 cells with control siRNA or PDCD4 siRNA. **(E)** Cell proliferation assays were performed 12, 24, 36, 48, 60 and 72 h after the transfection of SW480 cells with the control plasmid or PDCD4 overexpression vector. **(F and G)** Transwell assays were performed to evaluate the effect of PDCD4 on the migration of SW480 cells transfected with equal dose of control siRNA, PDCD4 siRNA, control plasmid or PDCD4 overexpression vector. F: representative images. G: quantitative analysis. **(H and I)** Apoptosis assays were performed 24 h after transfection of SW480 cells with equal dose of control siRNA, PDCD4 siRNA, control plasmid or PDCD4 overexpression vector. H: representative images; I: quantitative analysis.* $p < 0.05$; ** $P < 0.01$; *** $P < 0.001$.

Supplemental figure 2.

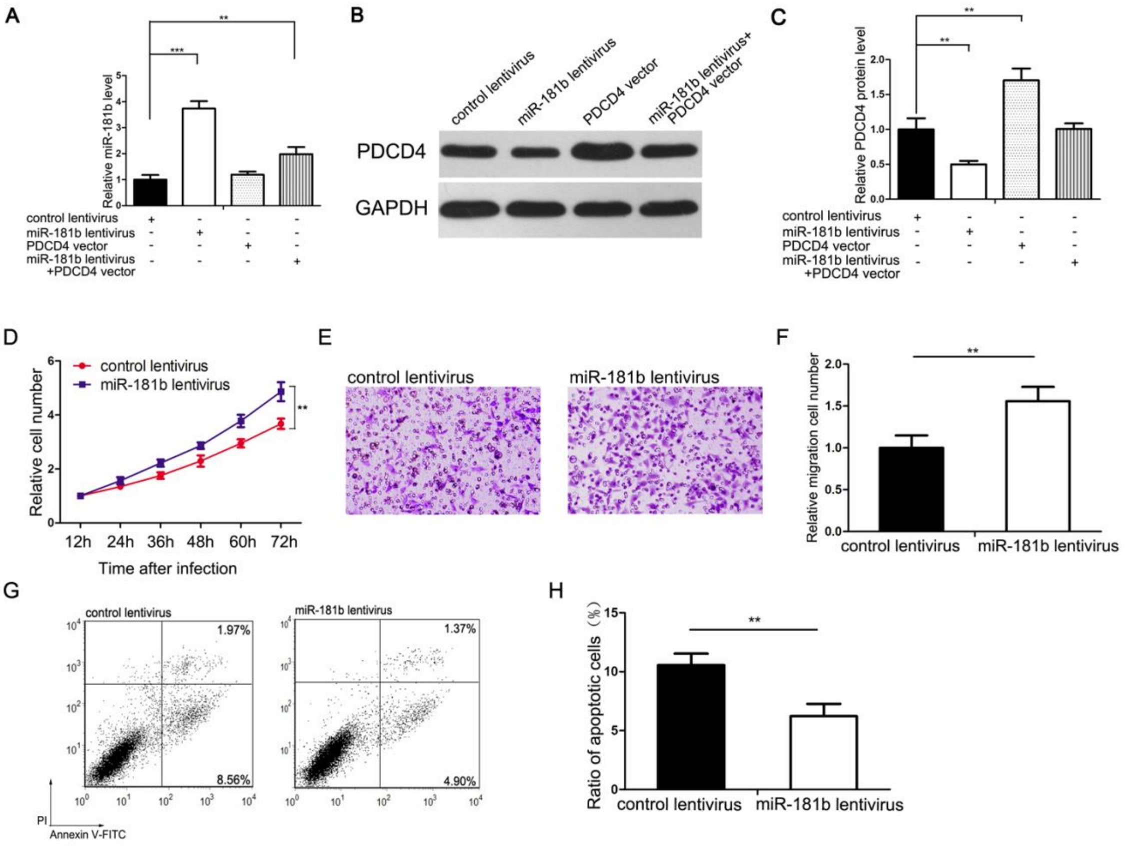

Supplemental Figure 2. Effects of miR-181b lentiviral infection on CRC cell proliferation, migration and apoptosis. (A) Quantitative RT-PCR analysis of miR-181b levels in SW480 cells that were infected with a control lentivirus or a lentivirus to overexpress miR-181b, or transfected with a PDCD4 overexpression plasmid, or co-transfected with a miR-181b overexpression lentivirus plus a PDCD4 overexpression plasmid. **(B and C)** Western blotting analysis of PDCD4 protein levels in SW480 cells that were infected with a control lentivirus or a lentivirus to overexpress miR-181b, or transfected with a PDCD4 overexpression plasmid, or co-transfected with a miR-181b overexpression lentivirus plus a PDCD4 overexpression plasmid. B: representative images; C: quantitative analysis. **(D)** Cell proliferation assays were performed 12, 24, 36, 48, 60 and 72 h after the infection of SW480 cells with a control lentivirus or a miR-181b overexpression lentivirus. **(E and F)** Transwell analysis of SW480 cells that were infected with a control lentivirus or a miR-181b overexpression lentivirus. E: representative images; F: quantitative analysis of the number of migrated cells. **(G and H)** Apoptosis assays were performed 24 h after infection of SW480 cells with a control lentivirus or a miR-181b overexpression lentivirus. G: representative images; H: quantitative analysis of the apoptotic cell ratio. ** $p < 0.01$; *** $p < 0.001$.
